# Carnosine/histidine-containing dipeptide supplementation improves depression and quality of life: systematic review and meta-analysis of randomized controlled trials

**DOI:** 10.1093/nutrit/nuae021

**Published:** 2024-03-27

**Authors:** Robel Hussen Kabthymer, Saeede Saadati, Mark Lee, Rohit Hariharan, Jack Feehan, Aya Mousa, Barbora de Courten

**Affiliations:** Department of Medicine, School of Clinical Sciences, Monash University, Melbourne, Australia; Department of Medicine, School of Clinical Sciences, Monash University, Melbourne, Australia; School of Health and Biomedical Sciences, RMIT University, Melbourne, Australia; Department of Medicine, School of Clinical Sciences, Monash University, Melbourne, Australia; Institute for Health and Sport, Victoria University, Melbourne, Australia; Monash Centre for Health Research and Implementation (MCHRI), Monash University, Melbourne, Australia; Department of Medicine, School of Clinical Sciences, Monash University, Melbourne, Australia; School of Health and Biomedical Sciences, RMIT University, Melbourne, Australia

**Keywords:** histidine-containing dipeptide, carnosine, depression, quality of life, meta-analysis

## Abstract

**Context:**

Mental ill-health is a common and growing issue, affecting 1 in 8 individuals or 970 million people worldwide in 2019. Histidine-containing dipeptides (HCDs) have been suggested to mitigate some aspects of mental ill-health, but a quantitative synthesis of the evidence is lacking. Therefore, a systematic review and meta-analysis of randomized controlled trials was conducted.

**Objective:**

To summarize the evidence on the effects of HCDs on mental health outcomes.

**Data Source:**

A systematic literature search was performed using electronic databases (Medline via Ovid, Embase via Ovid, Scopus, Google Scholar, and Cochrane) from inception to October, 2022.

**Data Extraction:**

Two authors independently extracted data using a structured extraction format.

**Data Analysis:**

Data analysis was performed using STATA version 17. Random-effects models were used, and heterogeneity was assessed using the I^2^ test. Quality appraisal was performed using the Cochrane risk-of-bias 2.0 tool and the Grading of Recommendations, Assessment, Development, and Evaluation (GRADE) approach.

**Conclusion:**

5507 studies were identified, with 20 studies fulfilling the inclusion criteria. Eighteen studies comprising 776 participants were included in the meta-analysis. HCD supplementation (anserine/carnosine, l-carnosine, β-alanine) caused a significant reduction in depression scores measured with the Becks Depression Inventory (−0.79; 95% CI: −1.24, -0.35; moderate certainty on GRADE) when compared with placebo. An increase in quality-of-life scores measured with the 36-item Short-Form survey (SF-36) (0.65; 95% CI: 0.00, 1.30) and low certainty on GRADE in HCDs (anserine/carnosine, l-carnosine, β-alanine) when compared with placebo were found. However, the rest of the outcomes did not show a significant change between HCD supplementation and placebo. Although the number of studies included in the meta-analysis was modest, a significant mean reduction was observed in depression score as well as an increase in quality-of-life score for the HCD group when compared with placebo. Most of the studies included had small sample sizes with short follow-up periods and moderate to high risk of bias, highlighting the need for further, well-designed studies to improve the evidence base.

**Systematic Review Registration:**

PROSPERO registration no. CRD42017075354.

## INTRODUCTION

In 2019, 1 in 8 individuals, or 970 million people worldwide, had a mental health disorder, with anxiety and depressive disorders being the most prevalent.^1^ Mental health disorders are currently the leading cause of disability-adjusted life-years (DALYs), with 418 million DALYs attributable to mental disorders in 2019 (16% of global DALYs).^2^ Various causes underpin the development and progression of mental health disorders. According to the Centers for Disease Control and Prevention, experiences related to other ongoing (chronic) medical conditions, such as cancer or diabetes, and biological factors or chemical imbalances in the brain are among the central causes of mental illnesses.^3^

The underlying pathophysiological mechanisms resulting in mental disorders are less well understood. Chronic low-grade inflammation has been observed in most mental disorders.^4–9^ In particular, low-grade inflammation has been proposed as a key underlying mechanism for mood disorders, including depression.^10–12^ Increased concentrations of biomarkers of low-grade inflammation such as C-reactive protein (CRP; >3 mg/L) have been identified,^4^ together with elevated levels of interleukin (IL)-6 and other inflammatory cytokines in blood and cerebrospinal fluid^5–9^ of patients with depression.

Histidine-containing dipeptides (HCDs) are a class of soluble peptides composed of histidine and an atypical amino acid. Different HCDs, such as carnosine, anserine, and balenine, result from variations in the structure of the dipeptide.^10^ Carnosine (β‐alanine, l‐histidine) is 1 HCD that has been researched extensively.^10^ Carnosine is a known exercise enhancer and has been utilized extensively in sports to enhance physical performance and muscle growth.^11^ Further, the presence of carnosine and its analogues in the brain suggests that these HCDs may play some physiological role in brain function, as endogenous antioxidants, neuromodulators, and neuroprotective molecules.^12^ Indeed, carnosine supplementation has been shown to affect behavior in several animal studies.^13–15^ Its capacity to reduce anxiety has been observed in rats,^15^ which has been attributed to lower cortisol levels.^16^ Interestingly, carnosine and its reverse structure, histidinyl-alanine, have also been shown to cause sedation and hypoactivity.^13–17^

In humans, carnosine supplementation has been found to enhance cognition and well-being.^18–20^ Dietary supplementation with carnosine also had beneficial effects on behavior in autistic children,^18^ and improved cognitive function in patients with schizophrenia.^19^ A recent study has shown that carnosine plus anserine supplementation improved cognitive function and physical capacity in older adults.^20^ Carnosine supplementation also led to significant improvements in quality of life for patients experiencing heart failure.^21^

Despite promising evidence of the beneficial effects of HCDs on mental health outcomes, the results from existing studies are inconsistent, and a comprehensive synthesis of evidence is lacking. Hence, the aim of this study was to systematically review and summarize the evidence regarding the effects of HCDs on mental health outcomes and to identify relevant evidence gaps.

## METHODS

The protocol for this review was prepared and registered on PROSPERO (CRD42017075354) and published previously.^22^ This systematic review conforms to the Preferred Reporting Items for Systematic Reviews and Meta‐Analyses (PRISMA) standards.^23^

### Data sources and searches

A systematic literature search was performed using electronic databases (MEDLINE via Ovid, Embase via Ovid, Scopus and Google Scholar, and Cochrane Library) from September 30 to October 20, 2022. The complete search strategy is available in Supplemental Table S1 (please see the Supporting Information online). Additional studies were identified by scanning bibliographies of relevant studies and systematic reviews discovered via the search method. Google was used to manually search for gray literature (studies not indexed in scientific databases). When the required data were not published, the relevant authors were contacted and the de-identified aggregate data for meta-analysis were requested.

### Study selection

Articles identified from the search strategy were considered eligible if they met the selection criteria outlined in a predetermined PICO (Population, Intervention, Comparison, Outcomes) framework shown in Table 1.

**Table 1:** PICOS criteria for inclusion of studies

Parameter	Criterion
P (Population)	Men or women, children or adults
I (Intervention)	HCDs (including the precursor of all HCDs [histidine] and precursor of carnosine [β‐alanine]) in different preparations, dosages, routes, or durations, alone or in combination with other interventions
C (Comparison)	Placebo or usual care or any pharmacological or nonpharmacological interventions (such as exercise, training, diet); placebo or standard care
O (Outcomes)	Measurement of psychological or mental health outcomes
S (Study type)	Randomized controlled trials (both parallel and crossover designs)
Language	Articles written in English only
Year	No restrictions to year of publication

*Abbreviation*: HCD, histidine-containing dipeptide.

The titles and abstracts of all records identified in the searches were imported into an online systematic review management platform (Covidence; Veritas Health Innovation Ltd). Two independent reviewers (R.H.K.and S.S.) examined the titles and abstracts, and eligible studies were retrieved for full-text review. Where there were disagreements on full text eligibility, these were resolved by conversation or by consulting a third reviewer (R.H.).

### Data extraction and quality assessment

Data were extracted by 2 independent reviewers (R.H.K. and S.S.) using a prespecified data-extraction spreadsheet. Data extracted included details of the study: first author, year of publication, country, study design, and sample sizes overall and in each arm of the trial; participants—age, comorbidities, body mass index; interventions—type, dose, duration, and frequency of the intervention and route of administration; and results—mean or median follow‐up value with standard deviations, standard errors, 95% confidence intervals (CIs), or interquartile ranges. All extracted data and computed data entries for meta‐analysis were cross‐checked for accuracy by multiple authors (R.H.K. and S.S.).

Two independent reviewers (R.H.K. and S.S.) assessed the risk of bias using the Cochrane risk-of-bias tool.^24^ Individual quality items were reviewed, including the randomization and allocation process; blinding of participants, investigators, and outcome assessors; prespecified selection criteria; dropout rates and statistical power and analysis methods; outcome assessment and reporting; and conflicts of interest of authors. Based on these items, each study was assigned a risk-of-bias rating of low, medium, or high (or insufficient information if a judgment was not possible due to lack of information). Disagreement was resolved through discussion or consideration by third reviewer (R.H.).

The quality of the evidence supporting each outcome was assessed using the Grading of Recommendations, Assessment, Development, and Evaluation (GRADE) methodology.^25^ Per GRADE standards, 2 reviewers (R.H.K. and S.S.) graded each outcome as high, moderate, low, or very low based on key criteria judged across the evidence, including risk of bias, heterogeneity (inconsistency), indirectness, and imprecision. Visual inspection of forest plots, consideration of the magnitude and direction of effect size estimates, and assessment of whether CIs overlapped and between-study variability were used to detect inconsistency. These factors were compared with the baseline values and cumulative supplement dose, which could logically explain inconsistency. Variations in the population, intervention, and outcomes of interest were considered for indirectness. The number of studies for a particular outcome, the pooled sample size, and the breadth of the CIs were used to determine the degree of imprecision.

### Data analysis

Statistical analyses were performed using Stata version 17 (StataCorp, College Station, TX, USA). Extracted data for aggregate outcome measures (post-supplementation) were pooled for meta‐analysis. Assuming clinical heterogeneity, data were analyzed using random-effects meta-analysis models to calculate the weighted mean differences (WMDs) between intervention and control groups at follow-up, with corresponding 95% CIs. Statistical heterogeneity was assessed using the *I^2^* test, with values of more than 50% indicating moderate to high heterogeneity. Studies with insufficient information to be pooled for meta‐analysis are presented using descriptive analysis. Sensitivity analyses were conducted where studies with a high risk of bias or having some concerns were excluded to assess their effects on the overall results.

## RESULTS

### Study characteristics

**Figure 1:**
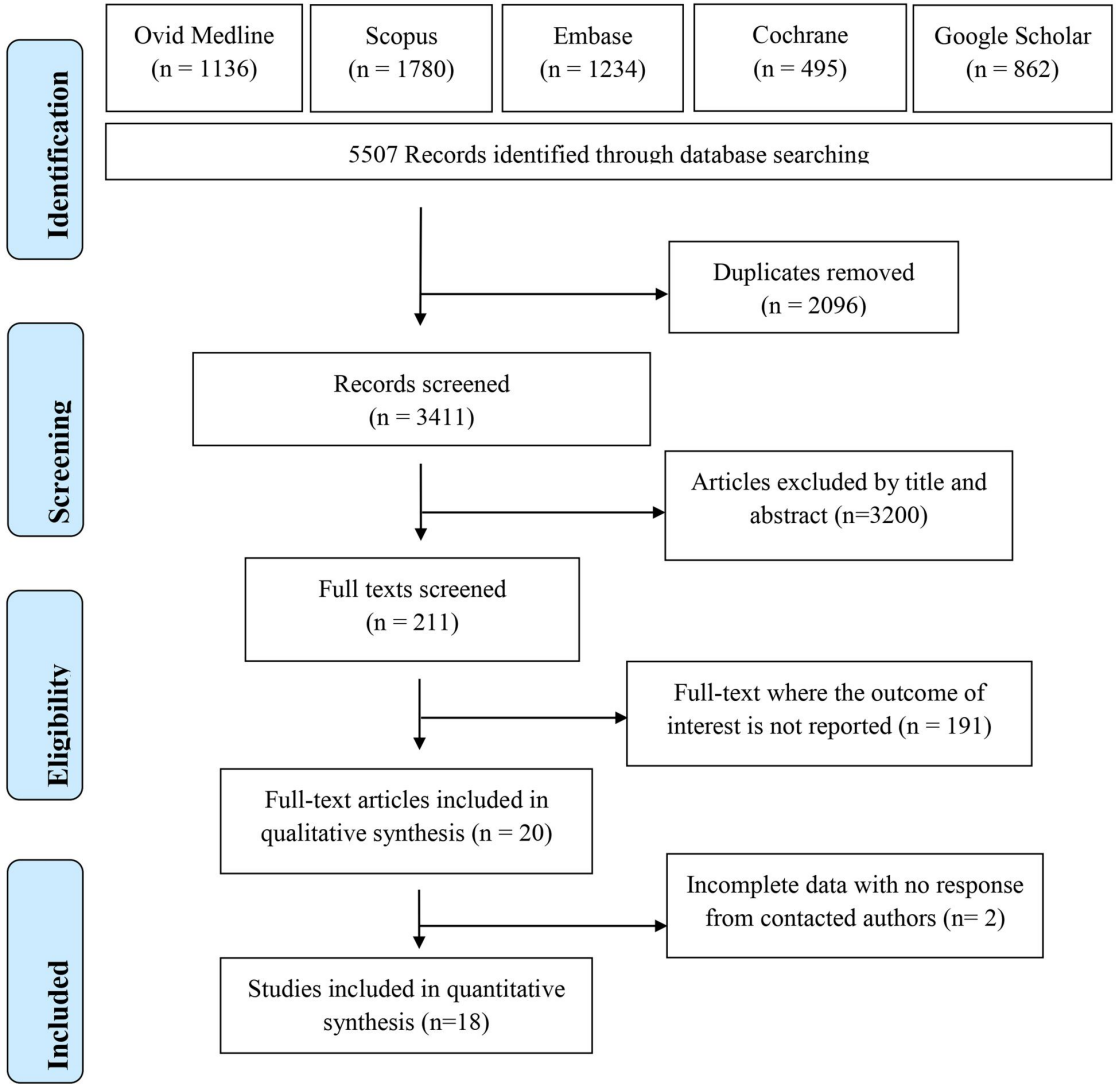
Flow diagram of the literature search process.

The search and screening process is presented in Fig. 1. Through the systematic search of 5 electronic databases, 5507 studies were identified, of which 2097 duplicates were excluded. Screening of titles and abstracts was completed for 3411 studies, resulting in 3200 further studies being excluded. The remaining 211 studies were screened by full text, with a further 191 studies excluded due to no outcome of interest being reported. In total, 20 studies were included in the review, of which 18 were pooled in meta-analysis. For the 2 studies excluded from meta-analysis, the authors were contacted using 3 e-mail attempts to provide the required data, but no response was received (Fig. 1).

**Figure 2:**
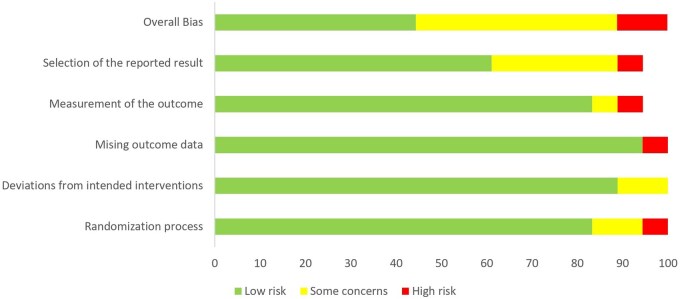
Summary of risk of bias for included studies.

Risk of bias was assessed for all included studies, of which 9 had a low risk of bias, 9 had moderate risk, and the remaining 2 studies had a high risk of bias (Table 2^18–21^^,^^26–41^ and Fig. 2).

**Table 2:** Summary of included studies for systematic review and meta-analysis of the effect of histidine-containing dipeptides on psychological outcomes

Reference	Study design	Population	Gender		Sample size, n	Age, y	BMI, kg/m^2^	Intervention and control		Duration	Pooled	Risk of bias
								Intervention	Control			
Sasahara et al, 2015^38^	Double-blind crossover RCT	Healthy	M		20	I = 51.9 ± 4.8 P = 51.0 ± 4.5 All = 51.5 ± 4.6	I = 26.0 ± 2.2 P = 24.9 ± 4.2 All = 25.5 ± 3.3	Histidine 1.65 g/d	Placebo	2 wk	Yes	Low
Araminia et al, 2020^29^	Double-blind RCT	Major depression	M and F		50	I = 34.76 ± 6.00 P = 32.11 ± 7.17	—	l-Carnosine 800 mg/d	Placebo	6 wk	Yes	Medium
Ghajar et al, 2018^31^	Double-blind RCT	Schizophrenia	M and F		51	I = 43.67 ± 8.78 P = 45.97 ± 9.30	—	2 g/d l-Carnosine	Placebo	8 wk	Yes	Medium
Szcześniak et al, 2014^20^	Double-blind RCT	Healthy	M and F		56	I = 81 ± 7.0 P = 80.5 ± 7.5	I = 28 ± 5 P = 27 ± 4	2.5 g/d Anserine/carnosine	Placebo	13 wk	Yes	Medium
Lombardi et al, 2015^21^	Open-label RCT	Heart failure	M and F		50	I = 61.2 ± 9.3 P = 62.3 ± 9.9	—	500 mg/d l-Carnosine	Standard care	6 mo	Yes	High
Varanoske et al, 2020^41^	Double-blind RCT	Healthy	M and F		19	I = 22.4 ± 3.0 P = 23.0 ± 3.8	—	12 g/d β-Alanine	Placebo	2 wk	Yes	Medium
del Favero et al, 2012^30^	Double-blind RCT	Healthy	M and F		18	I = 65 ± 4 P = 64 ± 7	I = 29.6 ± 2.7 P = 28.4 ± 4.3	3.2 g/d β-Alanine	Placebo	13 wk	Yes	Medium
Chez et al, 2002^18^	Double-blind RCT	Autism	M and F		31	I = 7.7 ± 2.4 P = 7.2 ± 2	—	800 mg/d Carnosine	Placebo	8 wk	Yes	Low
Ann Abraham et al, 2020^27^	Open-label RCT	Autism	M and F		67	I = 4.3 ± 0.7 P = 4.2 ± 0.5	I = 12.4 ± 4.8 P = 13.7 ± 4.2	10–15 mg/kg/d l-Carnosine	Standard care	2 mo	Yes	Medium
Allman et al, 2018^26^	Double-blind RCT	Parkinson disease	M and F		19	I = 68 ± 9 P = 68 ± 9	I = 27 ± 3 P = 26 ± 4	3.8 g/d β-Alanine	Placebo	4 wk	Yes	Medium
Mehrazad-Saber et al, 2018^37^	Double-blind RCT	Autism	M and F		43	I = 8.59 ± 2.77 P = 8.35 ± 2.76	I = 16.67 ± 4.3 P = 16.27 ± 3.45	500 mg/d Carnosine	Placebo	2 mo	Yes	High
Ghajar et al, 2018^32^	Double-blind RCT	ADHD	M and F		56	I = 9.12 ± 2.18 P = 8.28 ± 1.59	—	800 mg/d l-Carnosine	Placebo	8 wk	No	Low
Hajizadeh-Zaker et al, 2018^33^	Double-blind RCT	Autism	M and F		50	I = 8.24 ± 2.22 P = 7.90 ± 1.89	—	800 mg/d l-Carnosine	Placebo	10 wk	Yes	Low
Arabzadeh et al, 2017^28^	Double-blind RCT	Obsessive compulsive disorder	M and F		44	I = 33.45 ± 13.8 P = 30.45 ± 8.25	—	1 g/d l-Carnosine	Placebo	8 wk	No	Low
Hisatsune et al, 2016^34^	Double-blind RCT	Healthy	M and F		41	I = 67.8 ± 5.6 P = 70.6 ± 5.1	I = 21.6 ± 3.0 P = 21.0 ± 5.4	1 g/d Anserine carnosine	Placebo	3 mo	Yes	Low
Masuoka et al, 2019^36^	Double-blind RCT	Mild cognitive impairment (MCI)	M and F		50	I = 72.9 ± 8.8 P = 73.6 ± 6.1	I = 22.2 ± 2.8 P = 21.5 ± 2.6	1 g/d Anserine carnosine	Placebo	12 wk	Yes	Low
Varanoske et al, 2018^40^	Double-blind RCT	Healthy	M and F		40	I = 22.4 ± 3.0 P = 23.0 ± 3.8 All = 22.7 ± 3.3	—	12 g/d β-Alanine	Placebo	2 wk	Yes	Medium
Chengappa et al., 2012^19^	Double-blind RCT	Schizophrenia	M and F	70		I = 46.6 ± 8.5 P = 46.5 ± 9	—	l-Carnosine 500 mg/d at week 1 to 2 g/d at week 4	Placebo	4 wk	Yes	Low
Katakura et al, 2017^35^	Double-blind RCT	Healthy elderly	M and F	60		I = 60.4 ± 2.1 P = 65.3 ± 1.6	I = 21.0 ± 0.51 P = 21.1 ± 0.86	2 g/d Anserine/carnosine (3:1 ratio)	Placebo	3 mo	Yes	Low
Shirotsuki et al, 2017^39^	Open-label RCT	RCT	M and F	80		I = 35.44 ± 10.29 C = 38.35 ± 8.83	—	200 mg/d Histidine	Standard care	8 wk	Yes	High

*Abbreviations*: ADHD, attention-deficit/hyperactivity disorder; BMI, body mass index; C, control; F, females; I, intervention; M, males; P, placebo; RCT, randomized controlled trial.

Ten out of the 20 studies used carnosine,^18^^,^^19^^,^^21^^,^^27–29^^,^^31–33^^,^^37^ while the remaining studies used histidine (2 studies),^38^^,^^39^ anserine carnosine solution (4 studies),^20^^,^^34–36^ and β-alanine (4 studies).^26^^,^^30^^,^^40^^,^^41^ All studies used a parallel design, except for 1 study which used a crossover design.^38^ Two studies were open-label randomized controlled trials (RCTs)^21^^,^^39^ and all other studies used a double-blind procedure. Six studies were conducted in Iran,^28^^,^^29^^,^^31–33^^,^^37^ with the others conducted in the United States (5 studies),^18^^,^^19^^,^^26^^,^^40^^,^^41^ Japan (5 studies),^34–36^^,^^38^^,^^39^ Brazil (1 study),^30^ India (1 study),^27^ Italy (1 study),^21^ and Poland (1 study).^20^ All studies were published in English, with sample sizes ranging from 18 to 80 participants. Intervention durations ranged from 2 weeks to 6 months, with the majority of studies having durations of less than 2 months.

The mean age of participants ranged from 4.2 to 73.6 years. Of the 20 RCTs, 8 studies included healthy participants, while the rest included participants with comorbidities. These included autism spectrum disorder in 4 studies,^18^^,^^27^^,^^33^^,^^37^ schizophrenia in 2 studies,^19^^,^^31^ with single studies in major depressive disorder,^29^ mild cognitive impairment,^36^ heart failure,^21^ obsessive-compulsive disorder (OCD),^28^ Parkinson’s disease,^26^ and attention-deficit/hyperactivity disorder (ADHD).^31^ (Table 2).

### Risk-of-bias assessment

Assessments of the methodological quality of the included trials are presented in Fig. 2. Overall, 8 studies were identified as low risk of bias, 9 studies were identified as having some concerns, and 3 studies as having a high risk of bias.

### Summary and meta-analysis

#### Depressive and mood disorders

##### Depression

Six studies assessed the effects of HCDs on depression using 4 different scales, including the Becks Depression Inventory (BDI), the Hamilton Depression Scale, the Geriatric Depression Scale (GDS), and the Calgary Depression Scale. Meta-analysis was performed for the GDS and BDI only as the remaining measures were reported only in single studies.

**Figure 3:**
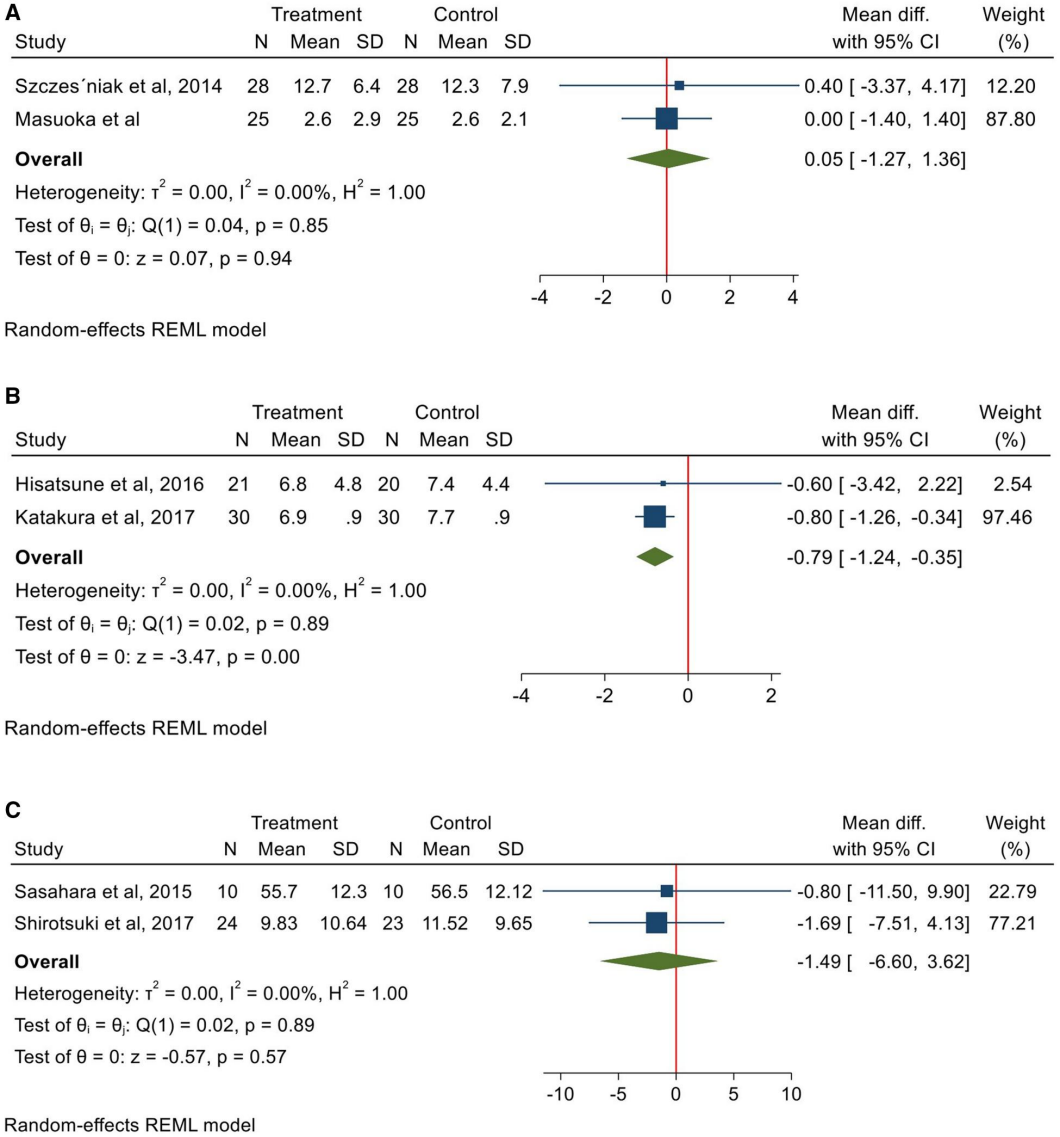

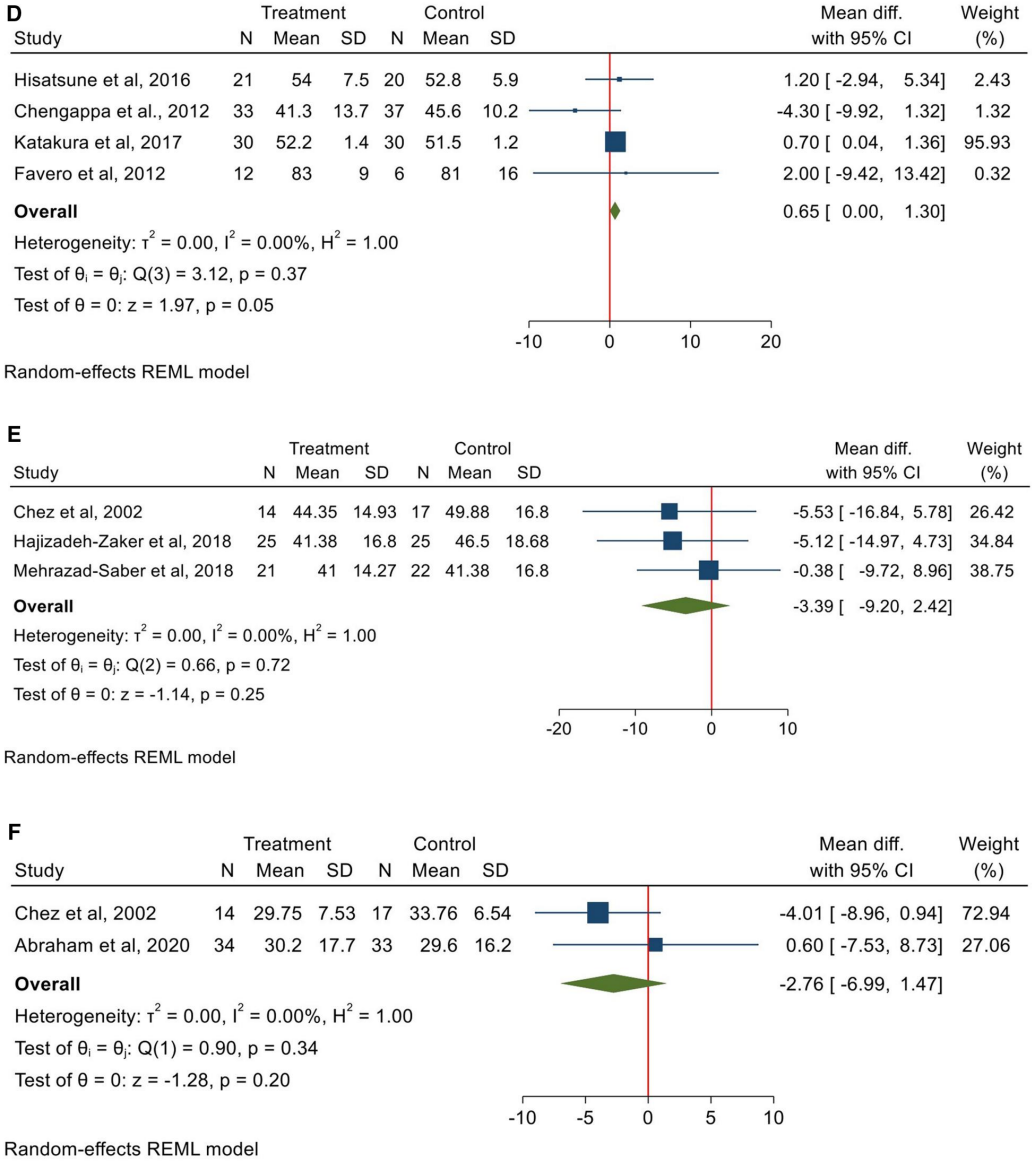
**Forest plots showing a meta-analysis of mean difference for mental health outcomes for the (A) Geriatric Depression Scale (GDS), (B) Becks Depression Inventory (BDI), (C) Profile of Mood States (POMS), (D) 36-item Short-Form Health Survey (SF-36), (E) Gilliam Autism Rating Scale (GARS), and (F) Childhood Autism Rating Scale (CARS)**. *Abbreviations*: diff., difference; REML, restricted maximum likelihood.

The GDS was used in studies by Szczesniak et al^20^ and Masuoka et al^36^ to examine symptoms of depression in elderly participants. Meta-analysis of the 2 studies including 106 participants showed no significant difference in mean GDS scores between the anserine/carnosine solution group and the placebo group (WMD = 0.05 [95% CI: −1.27, 1.36]; *P *=* *0.94; *P* for heterogeneity [*P*_het_] = 0.85, *I^2^* = 0%) (Fig. 3A).

Two studies assessed depression using the BDI.^34^^,^^35^ Meta-analysis of these 2 studies with 101 participants showed a significant reduction in BDI score in the carnosine/HCD group as compared with the placebo group (WMD = –0.79 [95% CI: –1.24, –0.35]; *P *=* *0.00; *P*_het_ = 0.89, *I^2^* = 0%). (Fig. 3B).

One study assessed the effect of l-carnosine or placebo as adjuvant treatment for 4 weeks in 70 patients with schizophrenia. The results showed no significant mean change in depression scores measured with the Calgary Depression Scale.^19^

Another study assessed the effect of l-carnosine or placebo for 6 weeks in 50 patients with major depression. Significant improvements in depression scores measured with the Hamilton Depression Scale were observed in the l-carnosine group as compared with the placebo group (WMD = 3.15; 95% CI: 0.45–5.84; *P *=* *0.023).^29^

##### Profile of Mood State

Three studies reported on mood using the Profile of Mood State (POMS) tool.^38^^,^^39^^,^^41^ However, only 2 studies were included in the meta-analysis because the study by Varanoske et al^41^ did not report a total score rather only subscales of the POMS scale. A meta-analysis of the POMS scores^38^^,^^39^ with a total of 67 participants did not show a significant mean change in the carnosine group when compared with the placebo groups (WMD = –1.49 [95% CI: –6.60, 3.62]; *P* = 0.57; *P*_het_ = 0.89, *I^2^* = 0%) (Fig. 3C).

#### Schizophrenia, attention-deficit/hyperactivity disorder, and obsessive-compulsive disorder

##### Schizophrenia

In a study by Ghajar et al,^31^ 51 patients with schizophrenia received either 2 g/d of l-carnosine or placebo for a period of 8 weeks. Based on the Positive and Negative Symptoms of Schizophrenia (PANSS) scale, there was a significant improvement in total PANSS score (2.13, 0.96–3.31) and a reduction in negative symptoms (1.47, 0.50–2.43) in the l-carnosine group as compared with the placebo group (*P* < 0.05) but no difference in positive symptoms.

Another study by Chengappa et al^19^ included 70 participants with schizophrenia supplemented with l-carnosine for 4 weeks, starting from 500 mg/d at week 1 to 2 g/d at week 4. No significant changes in PANSS scores were observed.

##### ADHD

A study that assessed the effect of 800 mg/d of l-carnosine for 8 weeks in 56 children with ADHD showed no significant effect as compared with placebo when measured using teacher and parent ADHD rating scales.^32^

##### OCD

One study assessed the effect of 1 g/d l-carnosine or placebo for 10 weeks as adjuvant to fluvoxamine for OCD in 40 patients.^28^  l-Carnosine was more effective in reducing the total and compulsive subscale score of the Yale‐Brown Obsessive Compulsive Scale, but there was no change in the obsessive subscale score of the Yale‐Brown Obsessive Compulsive Scale as compared with placebo.

#### Quality of life

Among the included studies, quality of life was assessed using the 36-item Short Form survey (SF-36). Four studies reported the effect of HCDs on quality of life using the SF-36.^19^^,^^30^^,^^34^^,^^35^ In a meta-analysis of these 4 studies including 189 participants, there was a positive change in the quality of life based on the SF-36 in the carnosine/HCD group as compared with the placebo group (WMD = 0.65 [95% CI: 0.00, 1.30]; *P *=* *0.05; *P*_het_ = 0.37, *I^2^* = 3.96%) (Fig. 3D). In a sensitivity analysis excluding 1 study having “some concern” of risk of bias,^39^ the result remained significant (WMD = 0.72 [95% CI: 0.07, 1.37]; *P *=* *0.02; *P*_het_ = 0.95, *I^2^* = 0%).

#### Autism spectrum disorder

Studies that assessed the effect of HCDs on autism spectrum disorder used either the Gilliam Autism Rating Scale (GARS) or Childhood Autism Rating Scale (CARS). Meta-analysis of 3 studies using the GARS^18^^,^^33^^,^^37^ including 124 participants showed no significant mean differences between the l-carnosine and placebo groups (WMD = −3.39 [95% CI: −9.20, 2.42]; *P *=* *0.25; *P*_het_ = 0.72, *I^2^* = 0%) (Fig. 3E).

Two studies utilizing the CARS,^18^^,^^27^ with meta-analysis of 98 participants showed no significant mean difference in scores between the carnosine and placebo groups (WMD = −2.76 [95% CI: −6.99, 1.47]; *P *=* *0.20; *P*_het_ = 0.32, *I^2^* = 0%) (Fig. 3F).

##### Publication bias

Based on visual inspection of funnel plots and Egger’s test, there was no indication of publication bias for POMS (*P* = 0.81), GDS (*P* = 0.33), BDI (*P* = 0.17), GARS (*P* = 0.43), CARS (*P* = 0.79), or the SF-36 (*P* = 0.49) (*see*  Fig. S1 in the Supporting Information online).

##### GRADE assessment

Certainty of evidence was assessed using the GRADE approach. The certainty of the POMS scale meta-analysis was low; it was down-graded due to the inclusion of studies with some concern (moderate risk of bias) and studies that were conducted in populations with different health conditions (serious indirectness).

The certainty of the BDI scale was graded as moderate due to the inclusion of studies with small sample sizes (serious imprecision), and the SF-36 was graded as low due to the inclusion of studies having some concern of risk of bias (serious risk of bias) and a different direction of estimates (serious inconsistency). However, results from the GARS meta-analysis were graded as low, and were down-graded due to the inclusion of studies having some concern of risk of bias (serious risk of bias) and wide CIs of estimates (serious imprecision).

Last, the CARS meta-analysis was graded as low due to the inclusion of studies having some concern of risk of bias (serious risk of bias) and a different direction of estimates (serious inconsistency) (*see*  Table S2 in the Supporting Information online).

## DISCUSSION

This is a comprehensive systematic review and meta-analysis on the effect of HCDs on mental health outcomes. Histidine-containing dipeptides improved depression and quality of life but had no impact on other mental health outcomes, including schizophrenia, OCD, ADHD, other mood disorders, and autism spectrum disorder.

A significant mean reduction in depression scores measured by the BDI in the carnosine group as compared with the placebo group was found. Similarly, quality of life measured with the SF-36 showed a significant increase in the carnosine group as compared with the placebo group. In contrast, no significant effects were observed in other mental health outcomes, including autism spectrum disorder measured using GARS and CARS, mood measured with POMS, and depression measured with the GDS and Hamilton Depression Scale. Data for the effects of HCDs on disorders such as schizophrenia, OCD, and ADHD were not amenable to meta-analysis, and showed mixed results in the literature. Animal studies have reported the anti-stress and anti-depressant effects of carnosine.^12^^,^^13^ This is supported by previous studies that reported that carnosine counteracts the reduction in spleen index and the quantity of spleen lymphocytes, including natural killer (NK) cells, which were observed to decrease stress in mice.^12^ A study assessing the effect of chicken breast extract or carnosine (1 of the major components of chicken breast extract) on immobility time, an index of depressive-like behavior, found that carnosine had a significant anti-depressant effect.^13^ As evident from the results of the present review, few human studies have examined the effects of HCDs on depression.^20^^,^^34–36^ The BDI is reported to be more sensitive in detecting small changes after treatment as compared with other scales used to monitor depression.^42^ In addition, the available studies are limited by small sample sizes with short durations and varying doses; hence, there is a need for further high-quality and adequately powered research to assess the impact of HCDs on mental health outcomes.

In this study, quality of life measured with the SF-36 showed a significant improvement in a meta-analysis of 4 studies. The quality of evidence was low. The components of SF-36 are physical or emotional problems, physical limitations, bodily pain, general mental health, fatigue or energy, social functioning, and general health.^43^ As reported in previous studies, the SF-36 is a good measure of mental health outcomes.^44^ The improved quality-of-life score by carnosine supplementation may be via its effect on mental health, especially depression.^44–46^

Putative mechanisms for the observed anti-depressant activity of carnosine might, first, be due to its downregulation of 3-methoxy-4-hydroxyphenylglycol, a major metabolite of norepinephrine, suggesting that carnosine could reduce norepinephrine activity in the hippocampus.^15^^,^^47^ Second, the carnosine effect might be via maintenance of telomere length,^48^ with previous research showing an association of telomere erosion with stress-related depressive disorders.^49^ In addition, the anti-oxidative,^50^ anti-glycating,^51^ and anti-inflammatory^52^ properties of carnosine demonstrated in murine and human cells may have played a significant role in mitigating the underlying pathogenesis of stress and depression.

Chronic low-grade inflammation was observed in most mental health disorders.^4–9^ For instance, raised levels of CRP (>3 mg/L) in patients with depression,^4^ together with elevated levels of IL-6 and other inflammatory cytokines in blood and cerebrospinal fluid in patients with depression,^5–9^ are indicators of low-grade systemic inflammation. The pathophysiology of mood disorders is possibly underpinned by inflammation, making inflammation a potential treatment target.^53^^,^^54^

Finally, this meta-analysis found no significant effects of HCDs on the POMS and autism scales. However, the reports from individual papers were inconsistent, and the number of studies included in the meta-analysis was sparse, with several study limitations precluding firm conclusions. The precise implications here are the need for future high-quality studies on various mental health outcomes.

### Strengths and limitations of the study

A comprehensive search strategy was used, with the inclusion of gray literature. In addition, meta‐analyses were conducted on several mental health outcomes, thereby providing a comprehensive overview of the effects of HCD supplementation on a range of outcomes, based on the available evidence to date without restrictions on year of publication.

Despite these strengths, this review is limited by the inclusion of only studies written in English and the small number of studies for almost all of the outcomes (≤4 studies). The risk of bias for the majority of studies was moderate. The quality of the evidence was low to moderate for all outcomes. Additionally, most of the included studies had small sample sizes with short follow-up periods, highlighting the need for further, well-designed research in this area. Furthermore, because none of the studies included long-term results, it was not possible to establish whether improvements in these psychological scales would result in sustained better mental health outcomes.

## CONCLUSION

This study summarizes the effects of HCDs on mental health outcomes based on the available evidence from randomized clinical trials. Significant mean differences in the HCD groups were observed in depression scores measured with the BDI and quality of life measured with the SF-36 as compared with placebo.

## Supplementary Material

nuae021_Supplementary_Data

## Supporting Information

The following Supporting Information is available through the online version of this article at the publisher’s website.

*Table S1* **Sample OVID-MEDLINE search strategy**

*Table S2*  **GRADE assessment of the effect of carnosine/HCDs on mental health outcomes meta-analyses**

*Figure S1*  **Funnel plots showing the publication bias among studies included for (A) GDS, (B) BDI, (C) POMS, (D) SF-36, (E) GARS, and (F) CARS**
