# Supplementary material for: Carnosine/histidine-containing dipeptide supplementation improves depression and quality of life: systematic review and meta-analysis of randomized controlled trials
Source: Nutr Rev. 2024 Mar 27;83(2):e54–64. doi: 10.1093/nutrit/nuae021 (PMC12013809; doi:10.1093/nutrit/nuae021)
Supplement: nuae021_Supplementary_Data [file nuae021_supplementary_data.zip › nuae021_Supplementary_Data/Supplementary file.docx]

| 1. Carnosine/ 2. carnosine.mp. 3. beta alanylhistidine.mp. 4. Anserine/ 5. anserine.mp. 6. beta alanyl 3 methylhistidine.mp. 7. ophidine.mp. 8. exp beta-alanine/ 9. beta alanine*.mp. 10. 3 aminopropionic acid.mp. 11. N-Acetyl-Carnosine.mp. 12. N-Acetyl-L-Carnosine.mp. 13. beta alanyl l histidine.mp. 14. beta-ala-his.mp. 15. l histidine beta alanyl.mp. 16. l alpha alanyl l histidine.mp. 17. histidine.mp. 18. balenine.mp. 19. or/1-18 20. randomi?ed controlled trial.pt. 21. controlled clinical trial.pt. 22. randomi?ed.ti,ab. 23. placebo.ti,ab. 24. clinical trials as topic.sh. 25. randomly.ti,ab. 26. trial.ti. 27. or/20-26 28. exp animals/ not exp humans/ 29. 27 not 28 30. Meta-Analysis as Topic/ 31. meta analy$.tw. 32. metaanaly$.tw. 33. Meta-Analysis/ 34. (systematic adj (review$1 or overview$1)).tw. 35. exp Review Literature as Topic/ | 1. or/30-35 2. cochrane.ab. 3. embase.ab. 4. (psychlit or psyclit).ab. 5. (psychinfo or psycinfo).ab. 6. (cinahl or cinhal).ab. 7. science citation index.ab. 8. bids.ab. 9. cancerlit.ab. 10. or/37-44 11. reference list$.ab. 12. bibliograph$.ab. 13. hand-search$.ab. 14. relevant journals.ab. 15. manual search$.ab. 16. or/46-50 17. selection criteria.ab. 18. data extraction.ab. 19. 52 or 53 20. Review/ 21. 54 and 55 22. Comment/ 23. Letter/ 24. Editorial/ 25. animal/ 26. human/ 27. 60 not (60 and 61) 28. or/57-59,62 29. 36 or 45 or 51 or 56 30. 64 not 63 31. 27 or 65 32. 19 and 66 33. limit 67 to humans |
| --- | --- |

**Supplemental Table 1.** Sample OVID-MEDLINE search strategy

Supplementary Table 2: **GRADE** (Grading of Recommendations, Assessment, Development and Evaluations) quality of evidence for the included studies

| **Outcome and scale** | **Number of studies** | **Number of participants** | | **Certainty assessment** | | | |  | **Favours** | **Certainty** |
| --- | --- | --- | --- | --- | --- | --- | --- | --- | --- | --- |
|  |  | **Intervention**  **N (%)** | **Placebo**  **N (%)** | **Risk of bias** | **Inconsistency** | **Indirectness** | **Imprecision** | **Effect, random  [95% CI]** |  |  |
| Mood | | | | | | | |  |  | |
| Profile of mood state (POMS) | 2 RCT | 34 (50.7%) | 33 (49.3%) | Serious ^1^ | Not serious | Serious ^3^ | Not Serious | MD -1.49 (-6.60, 3.62) | No difference | ⨁⨁◯◯ low |
| Depression | | | | | | | |  |  | |
| Geriatric depression scale (GDS) | 2 RCT | 53 (50%) | 53 (50%) | Serious ^1^ | Not serious | Serious ^3^ | Not serious | MD 0.05(-1.27, 1.36) | No difference | ⨁⨁◯◯ Low |
| Beck’s depression inventory (BDI) | 2 RCT | 51 (50.5%) | 50 (49.5%) | Not serious | Not serious | Not serious | Serious ^4^ | MD -0.79 (-1.24, -0.35) | Carnosine(lower with carnosine) | ⨁⨁⨁◯ Moderate^f^ |
| Quality of life | | | | | | | | | | |
| 36 item Short form survey scale (SF36) | 4 RCT | 96 (50.8%) | 93 (49.2%) | Serious ^1^ | Serious ^2^ | Not serious | Not Serious | MD 0.65 (0.00, 1.30) | Carnosine  (Higher with carnosine) | ⨁⨁◯◯ low |
| Autism spectrum disorder (ASD) | | | | | | | | | | |
| Gilliam autism rating scale (GARS) | 3 RCT | 60 (48.4%) | 64 (51.6%) | Serious ^1^ | Not serious | Not serious | Serious ^4^ | MD -3.39 (-9.20, 2.42) | No difference | ⨁⨁◯◯ low |
| Childhood autism rating scale (CARS) | 2 RCT | 48 (49%) | 50 (51%) | Serious | Serious ^2^ | Not serious | Not Serious | MD -2.76 (-6.99, 1.47) | No difference | ⨁⨁◯◯ low |

^1^ Downgraded once due to high or moderate risk of bias for some studies or outcomes

^2^ Downgraded once for inconsistency due to variations in effect estimate directions and/or CIs

^3^ Downgraded once for indirectness due to the use of different criteria and/or tools/ methods across included studies

4 Downgraded once for imprecision due to small sample size studies and/or wide CI

A) B)

C)

D) E)

F)

Supplementary figure 1: funnel plots showing the publication bias among studies included for A) GDS B) BDI C) POMS D) SF-36 E) GARS F) CARS
